# Two Cases of Symptomatic Familial Hypocalciuric Hypercalcemia: Treatment Response to Calcimimetic Therapy

**DOI:** 10.1210/jcemcr/luae096

**Published:** 2024-06-03

**Authors:** Jonathan Shakesprere, Ramsha Shafiq, Inderpreet Madahar, Hugh B Quinn, Yashan Thakkar, Adnan Haider

**Affiliations:** Department of Internal Medicine, West Virginia University School of Medicine, Morgantown, WV 26505, USA; Section of Endocrinology and Metabolism, Department of Internal Medicine, West Virginia University School of Medicine, Morgantown, WV 26505, USA; Department of Endocrinology, Diabetes and Metabolism, Corewell Health, St. Joseph, MI 49085, USA; Section of Endocrinology and Metabolism, Department of Internal Medicine, West Virginia University School of Medicine, Morgantown, WV 26505, USA; Department of Internal Medicine, West Virginia University School of Medicine, Morgantown, WV 26505, USA; Section of Endocrinology and Metabolism, Department of Internal Medicine, West Virginia University School of Medicine, Morgantown, WV 26505, USA

**Keywords:** cinacalcet, QTc interval, hypercalcemia, familial hypocalciuric hypercalcemia

## Abstract

Familial hypocalciuric hypercalcemia (FHH) is marked by mild to moderate hypercalcemia, normal-elevated serum PTH levels, and relative hypocalciuria. Cinacalcet, a calcimimetic therapy, has been reported to reduce symptom burden and serum calcium levels in FHH. We report 2 adult males with chronic hypercalcemia, with initial concerns for primary hyperparathyroidism. Urine calcium screening and genetic testing confirmed FHH in both patients. Shortened QTc normalized while on cinacalcet in the first patient and reductions in serum calcium and PTH levels without symptomatic hypercalcemia were noted in the second patient. Calcimimetic therapy can potentially be offered to FHH patients, particularly those with hypercalcemia symptoms, serum calcium levels >1 mg/dL (0.25 mmol/L) above normal or at risk of cardiac arrhythmias. Cinacalcet treatment was overall well tolerated and significantly reduced serum calcium and PTH levels in 2 adult FHH patients over time. Calcimimetic therapy has shown promise in managing persistent hypercalcemia and potential adverse events in FHH patients. Potential barriers include indefinite treatment, cost, and possible adverse effects.

## Introduction

Familial hypocalciuric hypercalcemia (FHH) is a rare, inherited disorder defined by mild to moderate PTH-dependent hypercalcemia and relative hypocalciuria. Decreased sensitivity to serum calcium (Ca) concentrations leads to dysregulated calcium-regulated PTH release by the parathyroids and decreased urinary calcium excretion by the kidneys (1). Its lifelong clinical course can range from asymptomatic to mild symptoms such as malaise and polydipsia to infrequent to severe manifestations of pancreatitis or chondrocalcinosis.

FHH must be distinguished from the more common primary hyperparathyroidism (PHPT) as both have overlapping clinical presentations in the setting of hypercalcemia and normal to elevated PTH levels (2). A 24-hour urinary calcium-creatinine collection demonstrating hypocalciuria of <100 mg/day (<2.5 mmol/d) suggests FHH but is not diagnostic; up to 20% to 30% of patients with PHPT can have low 24-hour urine calcium levels. The measurement of the fractional excretion of calcium (FEca) is of greater specificity. A ratio <0.01 can identify nearly 80% of FHH-affected individuals, while a ratio >0.02 is more suggestive of PHPT. Mutational analysis of *CaSR, AP2S1,* and *GNA11* genes can confirm FHH when clinical suspicion remains high or the FEca ratio is indeterminate (3).

The emergence of the calcimimetic agent cinacalcet has demonstrated usefulness in patients with *CaSR*-associated mutations (4). We report 2 patients with FHH who subsequently received cinacalcet for persistent hypercalcemia.

## Case Presentation

### Case 1

**Figure 1.**
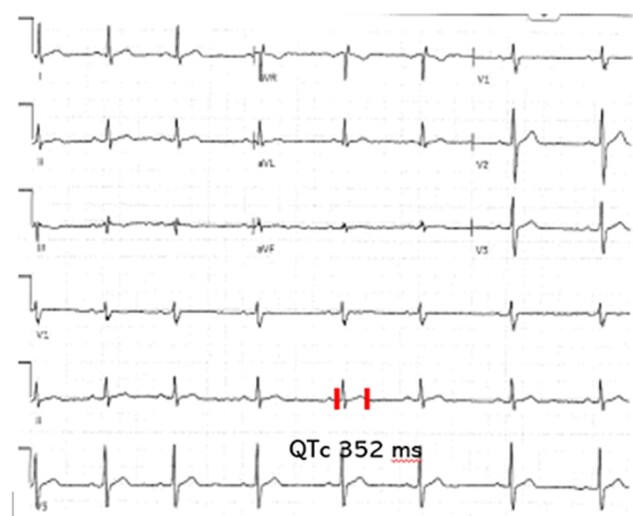
Electrocardiogram done during work-up of presyncope showing QTc 352 msec (2012).

A 55-year-old male was referred to the endocrinology clinic for evaluation of chronic hypercalcemia. Past medical history was significant for obesity, prediabetes, and hyperlipidemia. Eight years prior, he was evaluated for an episode of pre-syncope. Electrocardiogram at the time showed a shortened QTc interval (Fig. 1). Incidentally, he was also noted to have a serum Ca of 11.5 mg/dL [2.875 mmol/L, relative risk (RR): 2.12-2.62 mmol/L]. Laboratory work 1 month ago demonstrated a persistently elevated serum Ca of 11.5 mg/dL (2.875 mmol/L) with high serum intact PTH of 106 ng/L (RR: 10-65 ng/L).

The patient denied current symptoms of headache and abdominal pain as well as a history of bone fractures, nephrolithiasis, or endocrine gland tumors. No prior lithium or hydrochlorothiazide use was noted. Family history, though, was significant for PTH-dependent hypercalcemia in his biological mother and half-brother (same biological mother) as well as unspecified hypercalcemia in his biological sister. His half-brother had undergone unsuccessful subtotal parathyroidectomy with the removal of 3 parathyroid glands; his serum Ca remained elevated as high as 11.5 mg/dL (2.88 mmol/L) afterward.

### Case 2

A 64-year-old male was referred to the endocrinology clinic for continuation of PHPT management. The patient had previously been following up with a local endocrinologist and was prescribed cinacalcet 30 mg daily. Past medical history was significant for hypertension, type 2 diabetes mellitus, and depression. Routine laboratory work 1 month prior revealed an elevated serum Ca of 10.1 mg/dL (2.5 mmol/L) and a normal serum total 25-OH vitamin D level of 44.9 ng/mL (112.25 nmol/L, RR: 75-200 nmol/L).

He reported a long-standing history of hypercalcemia, previously undergoing a subtotal parathyroidectomy nearly 17 years ago after presenting with severe psychiatric symptoms in the setting of hypercalcemia to 12.9 mg/dL (3.2 mmol/L). Twenty-four-hour urine creatine-calcium collection at that time revealed urine calcium of 112 mg/24 hours (6.2 mmol/24 hours, RR: 2.50-7.50 mmol/24 hours). Sestamibi scan showed a left parathyroid adenoma; subsequent parathyroid surgical exploration revealed 4 gland hyperplasia, with 3.5 glands ultimately removed. However, his calcium levels only normalized briefly following surgery and were as high as 11.5 mg/dL (2.875 mmol/L) 48 hours postoperatively. No genetic testing was performed at that time. Cinacalcet treatment with 30 mg daily was started and maintained postoperatively.

He endorsed chronic polydipsia and depression and denied any history of osteoporosis or ancreatitis, as well as previous lithium use. No family history of hypercalcemia, parathyroid disease, or neck surgery was reported.

## Diagnostic Assessment

### Case 1

Repeat serum Ca (11.6 mg/dL, 2.97 mmol/L), ionized calcium (1.63 mmol/L, RR: 1.16-1.31 mmol/L), and PTH (144.2 ng/L) were all elevated, respectively (Table 1). A 24-hour urine calcium-creatinine collection was completed, showing a urine calcium excretion of 91.7 mg/24 hours (5.09 mmol/24 hours) and a FEca of 0.004 (Table 2). Confirmatory genetic testing using next-generation sequencing identified a heterozygous c.680G > A missense mutation in the *CaSR* gene consistent with FHH1.

**Table 1.** Patient baseline biochemical profile and response to cinacalcet therapy over time

Patient 1	Before Cinacalcet	Cinacalcet 30 mg daily
Serum calcium Normal range: 8.5–10.5 mg/dL; [2.12–2.62 mmol/L].	11.6 mg/dL [2.9 mmol/L]	10.5 mg/dL [2.62 mmol/L]
Serum ionized calcium Normal range: 4.64–5.24 mg/dL, [1.16–1.31 mmol/L]	6.52 mg/dL [1.63 mmol/L]	
Serum intact PTH Normal range: 10–65 pg/mL, [10–65 ng/L]	144.2 pg/mL 144.2 ng/L	61.9 pg/mL 61.9 ng/mL
Serum 25-OH, vitamin D Normal range: 20–50 ng/mL; [75–200 nmol/L]	21 ng/mL [52 nmol/L]	
Serum magnesium Normal range: 1.6–2.5 mg/dL; 0.85–1.10 mmol/L	2.3 mg/dL [0.9 mmol/L]	2.0 mg/dL [0.82 mmol/L]
Serum phosphorus (mg/dL) [mmol/L] Normal range: 2.4–4.1 mg/dL; 0.87–1.45 mmol/L	2.4 mg/dL [0.95 mmol/L]	2.6 mg/dL [0.78 mmol/L]

Patient 2	Cinacalcet 30 mg daily	Holding Cinacalcet	Cinacalcet 60 mg daily
Serum calcium Normal value: 8.5–10.5 mg/dL; 2.12–2.62 mmol/L	10.1 mg/dL [2.5 mmol/L]	(11.4 mg/dL [2.85 mmol/L	10 mg/dL [2.5 mmol/L]
Serum ionized calcium (mg/dL) [mmol/L] Normal range: 4.64–5.24 mg/dL, 1.16–1.31 mmol/L			5.56 mg/d [1.39 mmol/L]
Serum intact PTH Normal range: 10–65 pg/mL, [10–65 ng/L]		43.1 ng/L [43.1 pg/mL]	23.2 ng/L [23.2 pg/mL]
Serum 25-OH, vitamin D (ng/mL) [nmol/L] Normal range: 20–50 ng/mL; 75–200 nmol/L	70 ng/mL [174.3 nmol/L]		74 ng/mL [184 nmol/L]
Serum magnesium (mg/dL) [mmol/L] Normal range:1.6–2.5 mg/dL; 0.85–1.10 mmol/L	2.1 mg/dL [0.86 mmol/L]	2.0 mg/dL [0.82 mmol/L]	1.9 mg/dL [0.78 mmol/L]
Serum phosphorus (mg/dL) [mmol/L] Normal range: 2.4–4.1 mg/dL; 0.87–1.45 mmol/L	2.8 mg/dL [0.90 mmol/L]	3 mg/dL [0.96 mmol/L]	2.9 mg/dL [0.93 mmol/L]

**Table 2.** Patient 24-hour urine profiles and fractional excretion of calcium

Urine Testing Results	Patient 1	Patient 2
Urine calcium Normal range: Not reported	4.7 mg/dL [1.175 mmol/L]	3.7 mg/dL [1.17 mmol/L]
Urine calcium/specimen Normal range: 100–300 mg/day; 2.5–7.50 mg/day	91.7 mg/dL [2.29 mmol/L]	44.4 mg/dL [1.11 mmol/L]
Urine creatinine (mg/dL) [mmol/L] Normal range: Nor reported.	86 mg/dL [7.60 mmol/L]	124 mg/dL 10.96 mmol/L]
Urine creatinine/specimen (mg/day) [mmol/day] Normal range: 500–2000 mg/day; 4420–17 680 mmol/day	1677 mg/dL [148 mmol/L]	1488 mg/dL 131.5 mmol/L]
Urine collection volume Normal range: 800–2000 mL; 0.8–2.0 L	1950 mL 1.95 L	1200 mL 1.2 L
Urinary calcium: creatinine clearance ratio Normal range: 0.01–0.02.	0.004	0.0026

### Case 2

Due to a history of early-onset 4 gland parathyroid hyperplasia, MEN1 and hyperparathyroid jaw-tumor syndrome were considered in the differential diagnosis. Anterior pituitary hormone levels were screened for and unremarkable. Cinacalcet was then held for 1 week, with repeat biochemical testing showing persistent hypercalcemia of 11.5 mg/dL (2.875 mmol/L) with normal PTH of 43.1 ng/L. A 24-hour urine calcium-creatinine collection was repeated and notably showed urine calcium of 44.4 mg/24 hours (2.48 mmol/24 hours) and an FEca of 0.0026. With suspicion of underlying FHH, genetic testing was ordered using next-generation sequencing and identified a heterozygous c.43C > T > A missense mutation in the *APS21* gene consistent with FHH3.

## Treatment

### Case 1

The patient was subsequently started on cinacalcet 30 mg daily. He tolerated calcimimetic treatment without adverse effects or episodes of symptomatic hypercalcemia.

### Case 2

The patient was resumed on cinacalcet 30 mg daily to monitor serum Ca and PTH response.

At his 6-month follow-up visit, the patient reported new right lower extremity shin pain, reproducible on palpation and without preceding falls or trauma. X-ray of the right tibia-fibula showed chondrocalcinosis without acute fracture; serum bone alkaline phosphatase was not suggestive of metabolic bone pathology. Serum and ionized Ca remained elevated at 11.1 mg/dL (2.77 mmol/L) and 1.39 mmol/L, respectively, with normal serum PTH of 23.3 ng/L and 25-OH Vit D of 74 ng/mL (185 nmol/L). Cinacalcet was subsequently increased to 60 mg daily.

## Outcome and Follow-up

### Case 1

**Figure 2.**
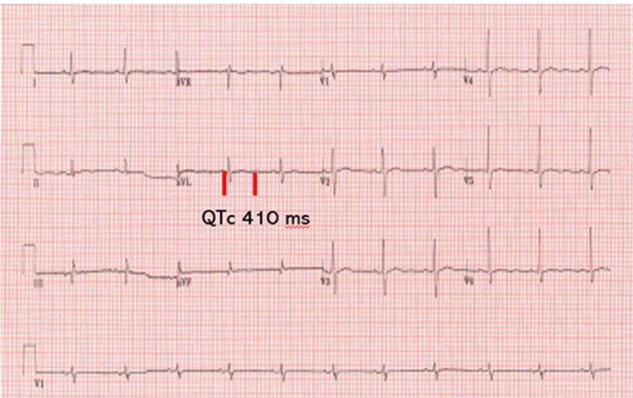
Repeat electrocardiogram done after 3 weeks of cinacalcet treatment QTc 410 msec (2024).

Repeat Ca and PTH levels completed at a 3-month follow-up visit showed improvement in calcium levels as well as normalization of his QTc interval (Table 1, Fig. 2). Cinacalcet was then increased to 60 mg daily but, due to subsequent persistent nausea, was reduced back to the 30 mg dose.

### Case 2

Monitoring lab work at the next follow-up visit 9 months later showed a reduction of serum Ca to 10.3 mg/dL (2.57 mmol/L). Repeat serum Ca 2 months later by his primary care provider showed Ca normalization to 9.5 mg/dL (2.37 mmol/L). At present, the patient is scheduled for an annual endocrine follow-up and has been tolerating cinacalcet treatment at 60 mg daily without adverse effects.

## Discussion

FHH is characterized by chronic mild to moderate hypercalcemia, normal to elevated serum PTH levels, and low urinary calcium excretion. It is associated with 3 autosomal dominant loss-of-function mutations impairing the extracellular Ca-sensing receptor (CaSR) or its associated proteins (5). FHH1, comprising >60% of cases, results from missense mutations in the *CaSR* gene impairing receptor-Ca affinity and binding. FHH2 is linked to dysfunction of the downstream G-protein subunit a_11_ (GNA11) regulating appropriate PTH release, while altered receptor-mediated endocytosis and signal transduction by adaptor-related protein complex 2, sigma 1 subunit (AP2S1) occurs in FHH3.

Moderate to high hypercalcemia, irrespective of etiology, can block sodium movement through voltage-gated sodium channels and cause reduced depolarization and abnormal action potentials (6). Shortened QTc intervals (<360 ms, RR: 360-460 ms), an electrocardiographic manifestation of early cardiac repolarization, were documented in 3 family members with FHH type 1. As a consequence of hypercalcemia, this shortening of the cardiac action potential can lead to fatal arrhythmias and sudden cardiac death (7). Interestingly, the use of cinacalcet in these 3 family members with FHH-1 led to rectification of the QTc interval. CaSR is expressed in cardiac myocytes, especially in the left ventricle myocardium, where it exerts membrane-stabilizing effects (8). The latter is further suggested by the anti-arrhythmic properties of putrescine, a natural ligand of CaSR (9).

An allosteric modulator that increases extracellular Ca sensitivity and expression of the CaSR in the parathyroids and kidneys, cinacalcet has previously been shown to decrease serum Ca levels and PTH secretion in PHPT as well as secondary hyperparathyroidism from chronic kidney disease. Cinacalcet has also been reported to improve symptoms and decrease serum Ca in FHH as well as neonatal severe hyperparathyroidism. Timmers et al first reported calcium normalization over 12 months in 2006 with maintenance cinacalcet use (10). Mayr et al's review of 16 case reports in 2016 reported a majority showing successful cinacalcet treatment of FHH as assessed by reductions in both symptoms and serum Ca levels (11). Rasmussen et al demonstrated long-term biochemical improvements in serum Ca and PTH levels with well-tolerated, fixed-dose cinacalcet treatment over 3 years (6). Furthermore, Howles et al found benefits from treating the rare AP2S1 mutation-related FHH3 with calcimimetic (12).

Calcimimetic therapy can potentially be offered to patients with FHH, particularly those with symptomatic hypercalcemia, serum Ca level >1 mg/dL (>0.25 mmol/L) above the upper limit of normal. Severe complications such as recurrent pancreatitis or inflammatory arthritis are further possible indications. FHH patients with syncope and documented short QTc intervals can now also be considered candidates for cinacalcet treatment (7). Potential adverse effects include abdominal discomfort, nausea, and vomiting. Extended daily treatment up to 3 years has already been shown to be well-tolerated; thus, even longer-term treatment with monitored surveillance can reasonably be expected to be tolerated.

Our decision to treat both patients with daily cinacalcet was based on a history of persistently elevated Ca levels >1 mg/dL (>0.25 mmol/L) above normal. Although an increased dose resulted in adverse effects for the first patient, both patients have thus far tolerated daily cinacalcet therapy without symptomatic hypercalcemia. Of note, the normalization of serum Ca in the second patient, who tolerated 60 mg daily, underlies the potential long-term benefits of calcimimetic. Although further time is needed to assess continued cinacalcet response and tolerance, our cases illustrate its potential efficacy and utility in FHH patients. Potential barriers include lifelong treatment as well as cost and potential adverse effects.

## Learning Points

- Distinguishing FHH from primary hyperparathyroidism involves family history, age of onset of disease, and determination of the calcium-to-creatinine clearance ratio, but only a genetic diagnosis is definitive.
- Screening for short QTc can be done in FHH patients as hypercalcemia can predispose patients to syncope, presyncope, and potential life-threatening arrhythmias.
- Evaluating FHH patients when serum Ca is greater than 1 gm/dl (> 0.25 mmol/L) above the upper limit of normal for hypercalcemia symptoms and screening for serious conditions such as short QTc intervals can help assess cinacalcet therapy as a treatment option.
- FHH-associated moderate hypercalcemia in 2 adult patients showed that calcimimetic therapy can be well tolerated and efficacious in reducing serum Ca and parathyroid hormone levels.

## Data Availability

Original data generated and analyzed for this case report are included in this published article.
